# Effect of Different Fertilizer Types on Quality of Foxtail Millet under Low Nitrogen Conditions

**DOI:** 10.3390/plants13131830

**Published:** 2024-07-03

**Authors:** Tingting Zheng, Shu Wang, Mengyao Wang, Jiao Mao, Yuanmeng Xu, Jiangling Ren, Yuhan Liu, Sichen Liu, Zhijun Qiao, Xiaoning Cao

**Affiliations:** 1 Center for Agricultural Genetic Resources Research, Shanxi Agricultural University, Taiyuan 030031, China; zhengtingting056@163.com (T.Z.); sj917000@163.com (S.W.); wangmengyao0113@163.com (M.W.); maojiao6958@126.com (J.M.); xuyuanmeng1229@163.com (Y.X.); renjiangling0@163.com (J.R.); liuyuhan202305@163.com (Y.L.); lsch209@163.com (S.L.); 2 College of Agriculture, Shanxi Agricultural University, Jinzhong 030801, China; 3 Key Laboratory of Crop Gene Resources and Germplasm Development in Loess Plateau, Ministry of Agriculture and Rural Affairs, Taiyuan 030031, China

**Keywords:** agronomic traits, fertilization type, foxtail millet, starch pasting, quality

## Abstract

In order to clarify the effect of different fertilizers on foxtail millet quality under low nitrogen conditions, we used JGNo.21 and LZGNo.2 as experimental materials and set up five treatments, including non-fertilization, nitrogen, phosphorus, compound, and organic fertilizers, to study the regulation of different fertilizer types on agronomic traits, nutrient fractions, and pasting characteristics of foxtail millet under low nitrogen conditions. Compared with the control, all of the fertilizers improved the agronomic traits of JGNo.21 to a certain extent. Nitrogen and compound fertilizer treatments reduced the starch content of JGNo.21; the starch content was reduced by 0.55% and 0.07% under nitrogen and compound fertilizers treatments. Phosphorus and organic fertilizers increased starch content, and starch content increased by 0.50% and 0.56% under phosphorus and organic fertilizer treatments. The effect of each fertilizer treatment on protein content was completely opposite to that of starch; different fertilizer treatments reduced the fat content of JGNo.21 and increased the fiber content. Among them, nitrogen and phosphorus fertilizers increased the yellow pigment content; the yellow pigment content increased by 1.21% and 2.64% under nitrogen and phosphorus fertilizer treatments. Organic and compound fertilizers reduced the content of yellow pigment; the yellow pigment content was reduced by 3.36% and 2.79% under organic and compound fertilizer treatments. Nitrogen and organic fertilizers increased the fat content of LZGNo.2; the fat content increased by 2.62% and 1.98% under nitrogen, organic fertilizer treatment. Compound and phosphorus fertilizer decreased the fat content; the fat content decreased by 2.16% and 2.90% under compound and phosphorus fertilizer treatment. Different fertilizer treatments reduced the cellulose and yellow pigment content of LZGNo.2. The content of essential, non-essential, and total amino acids of JGNo.21 was increased under compound and nitrogen fertilizer treatments and decreased under organic and phosphorus fertilizer treatments. The content of essential, non-essential, and total amino acids of LZGNo.2 was significantly higher under compound, nitrogen, and organic fertilizer treatments compared with control and significantly decreased under phosphorus fertilizer treatments. Nitrogen and compound fertilizer treatments significantly reduced the values of peak viscosity, trough viscosity, breakdown viscosity, final viscosity, setback viscosity, and pasting time of each index of JGNo.21; phosphorus and organic fertilizer treatments improved the values of each index. In contrast, the pasting viscosity of LZGNo.2 increased under phosphorus fertilizer treatment and decreased under nitrogen fertilizer treatment. Reasonable fertilization can improve the quality of foxtail millet, which provides a scientific theoretical basis for improving the quality of foxtail millet.

## 1. Introduction

Foxtail millet (*Setaria italica* L.) is one of the ancient crops that originated in China and is a main crop in farming culture [1,2]. Foxtail millet has the characteristics of strong drought resistance, short growth cycle, high photosynthetic efficiency, and high water use efficiency [3], which make them ideal model plants for studying stress tolerance mechanisms and crop quality effects in crops [2,4]. Foxtail millet not only contains starch, protein, fat, dietary fiber, and vitamins but also contains rich functionally active substances, such as phenols, carotenoids, and so on [5]. Many factors influence the quality of millet, including variety, fertilization, environment, etc., among which fertilization is the key factor affecting the quality of millet.

Fertilizer application is an important measure to improve crop quality, and it is beneficial for improving soil fertility and achieving a virtuous cycle in the farming system [6]. Nitrogen fertilizer application can significantly increase dry matter accumulation and nitrogen uptake, increase pre-flowering nitrogen storage in plant nutrient organs and delay their senescence [7], and increase the activities of leaf nitrate reductase, seed glutamine phthalimide synthase and glutamate synthase [8,9], which promotes nitrogen re-transportation from the plant, and consequently, improves the quality of the crop [10,11]. Inadequate nitrogen supply adversely affects plant height, leaf size, and photosynthesis [12]. Nitrogen level significantly affects the structure and physicochemical properties of starch granules; with increasing nitrogen level, the size of starch granules, the peak viscosity, and pasting temperature decreases, and the enthalpy of pasting increases [13,14]. Phosphorus fertilizer promotes chlorophyll synthesis, reduces the rate of chlorophyll degradation in the later stages of crop fertility, delays leaf senescence, and promotes crop dry matter accumulation [15,16]. Organic fertilizer can regulate crop photosynthesis by increasing chlorophyll content, leaf net photosynthetic rate, and stomatal conductance. In addition, organic fertilizer can increase the content of fat and protein in maize under drought stress [17]. Zhou et al. showed that organic fertilizer could increase the pasting viscosity and disintegration value of rice starch, reduce the pasting temperature and pasting enthalpy, and increase the content of essential amino acids [18]. Compound fertilizers reduce NH_3_ emissions by promoting soil nitrification, thereby increasing soil inorganic N content and improving leaf area index and N use efficiency [19]. Rational fertilization practices can improve crop quality.

However, although fertilizers have the potential to significantly increase grain quality, their indiscriminate use can lead to deterioration in soil structure, soil acid–base imbalance, and destruction of soil granular structure, which leads to a series of ecological problems such as soil compaction, salinization, and accumulation of nitrates in groundwater [20,21,22]. Excessive fertilizer inputs in current agricultural production are mainly manifested as excessive nitrogen application. Excessive application of N not only leads to increased costs of cultivation but also reduces the enrichment of beneficial microbial communities within crop grains, resulting in a decrease in crop quality [20,23]. Therefore, optimizing fertilizer management is essential to achieve sustainable agricultural development.

Nitrogen-reduced fertilization can increase the crystallinity, swelling power, and enthalpy of pasting of wheat starch granules and reduce the pasting temperature [24]. Reducing nitrogen fertilization can effectively improve nitrogen metabolism and optimize dry matter accumulation and distribution [25], which affect protein content [24]. Reducing nitrogen fertilizer application can improve nitrogen utilization [26]. In addition, nitrogen-reduced fertilization can reduce nitrous oxide emissions and achieve a good match between environmental production and sustainable agricultural development goals [27].

This study aims to clarify the effects of different fertilizer types on the agronomic traits, nutrient composition, and starch pasting properties of foxtail millet on the basis of long-term low nitrogen conditions, with a view to elucidating the regulatory mechanism of foxtail millet phenotypes and quality traits under low nitrogen conditions, revealing the intrinsic relationship between different fertilizer types and the quality of foxtail millet, and providing theoretical basis for rational fertilization and high-yield and high-quality cultivation techniques of foxtail millet.

## 2. Results

### 2.1. Effect of Different Fertilizer Treatments on Agronomic Traits of Foxtail Millet

As shown in Table 1, the responses of agronomic traits to different fertilization treatments varied. Compared with the control, all the fertilization treatments favored the improvement of agronomic traits of JGNo.21, with plant height, spike length, stem thickness, spike weight, and grain weight increased to different degrees. Plant height varied from 146.11 to 161.89 cm, with increases of 8.67%, 3.19%, 10.80%, and 2.46% under JN, JS, JC, and JP treatments, respectively. Spike length varied from 20.63 to 23.11 cm, with increases of 9.32%, 12.01%, 10.12%, and 4.65% under JN, JS, JC, and JP treatments, respectively. Spike weight varied between 14.75 and 22.76 g, grain weight varied between 13.48 and 16.79 g, and the order of both was JS > JN > JP > JC > JCK under different treatments. Under JS treatment, the increase in spike length, spike weight, and grain weight of JGNo.21 was relatively large, indicating that the compound fertilizer treatments had a greater impact on the grain spike traits. Stem thickness varied from 7.11 to 8.10 cm, of which the larger increases were in the JN treatment and the smaller increases were in the JP treatment, indicating that the nitrogen fertilizer treatment had a larger effect on the stem traits of the foxtail millet.

Compared with JGNo.21, the response of each agronomic trait of LZGNo.2 to fertilization treatments was different. The variation in plant height was 108.33–114.67 cm, which was increased to a certain extent under ZN, ZS, and ZC treatments and decreased under ZP treatment. The variation in spike length was 21.72–23.78 cm, in which the effect of fertilizer application was better under ZN and ZP treatments than ZS and ZC treatments, and compared with the control group, the decreases under ZS and ZC treatments were 6.01% and 3.85%, respectively. The effect of ZN and ZC treatments on enhancing stem thickness was more pronounced, with an increase of 8.94% and 3.58%, respectively, while stem thickness was reduced to some extent under ZS and ZP treatments. The variability in spike weight was 17.00–21.65 g. The increase in spike weight was 19.02% under ZN treatment. Grain weight varied from 13.10 to 16.91 g, and ZN treatment significantly increased grain weight, and the relationship of grain weight among different treatments was ZN > ZCK > ZP > ZS > ZC. In addition, varieties had a significant effect on plant height and stem thickness, and the plant height of JGNo.21 was generally better than that of LZGNo.2.

### 2.2. Effect of Different Fertilizer Treatments on the Nutritional Components of Foxtail Millet

**Figure 1:**
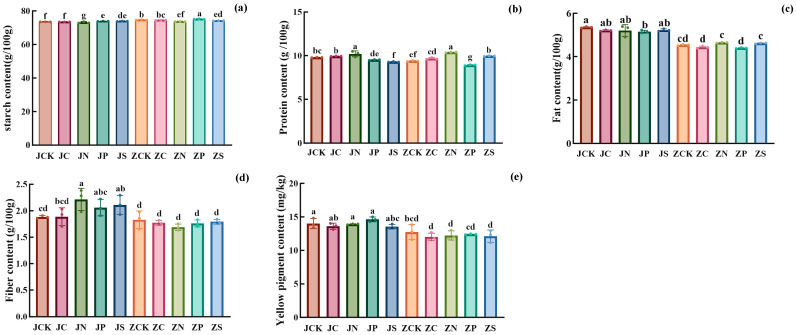
The effect of different fertilizer treatments on nutritional components: (**a**) Starch content; (**b**) Protein content; (**c**) Fat content; (**d**) Cellulose content; (**e**) Yellow pigment content. Different lowercase letters on the bar chart indicated that there were significant differences between different treatments (*p* < 0.05). JN: JGNo.21 nitrogen fertilizer group; JP: JGNo.21 phosphorus fertilizer group; JC: JGNo.21 compound fertilizer group; JS: JGNo.21 organic fertilizer group; JCK: JGNo.21 control group, no fertilization; ZN: LZGNo.2 nitrogen fertilizer group; ZP: LZGNo.2 phosphorus fertilizer group; ZC: LZGNo.2 compound fertilizer group; ZS: LZGNo.2 organic fertilizer group; ZCK: LZGNo.2 control group, no fertilization.

Compared with the control group (Figure 1), JP and JS treatments significantly increased the starch content, and JN treatment significantly decreased the starch content. The order of starch content of JGNo.21 under different fertilizer treatments was JS > JP > JCK > JC > JN; the fat content ranged from 5.15 to 5.36 g/100 g. Different fertilization treatments reduced the fat content, and there was no significant difference between groups. Compared with JCK, JC, JN, JP, and JS decreased by 2.80%, 2.80%, 3.79%, and 2.49%, respectively. The variation in protein content ranged from 9.30 to 10.20 g/100 g, and the order of protein content between different fertilization treatments was JN > JC > JCK > JP > JS. Compared with the control, the protein content increased by 4.20% and 1.34% in JN and JC treatments and decreased by 2.78% and 5.05% in JP and JS treatments; the protein content of different fertilization treatments was also increased. All the different fertilization treatments increased the cellulose content of JGNo.21. Among them, the increase under JN treatment was 17.39%. The content of the yellow pigment in each treatment group was 13.53–14.37 mg/kg, with the highest yellow pigment content under JP treatment and the lowest under JS treatment. Compared with JCK, the content of yellow pigment increased by 1.21% and 2.64%, respectively, under JN and JP treatments, while the content of yellow pigment decreased by 3.36% and 2.79%, respectively, under JS and JC treatments.

The starch content was significantly lower under ZN and ZS treatments and significantly higher under ZP treatment, and the order of starch content between different fertilization treatments was ZP > ZCK > ZC > ZS > ZN. The fat content of the LZGNo.2 was lower, and the fat content of each treatment ranged from 4.40 to 4.65 g/100 g, compared with ZCK, and increased by 2.62% and 1.98% under ZN and ZS treatments, while decreased by 2.16% and 2.90% under ZC and ZP treatments, respectively. Protein content ranged from 8.93 to 10.39 g/100 g, and the order of protein between different fertilization treatment groups was ZN > ZS > ZC > ZCK > ZP. Compared with ZCK, protein content under ZN, ZS, and ZC treatments increased by 10.63%, 5.93%, and 2.87% and decreased by 4.87% in the ZP treatments. Different fertilization treatments reduced the cellulose content of LZGNo.2, which decreased by 3.16%, 7.83%, 3.83%, and 2.26% in ZC, ZN, ZP, and ZS treatments, respectively. Different fertilization treatments reduced the yellow pigment content, and the order of yellow pigment content was ZCK > ZP > ZN > ZS > ZC.

### 2.3. Effects of Different Fertilizer Treatments on the Amino Acid Composition of Foxtail Millet

From Table 2, it can be seen that the EAA (essential amino acids) content under each fertilizer treatment in JGNo.21 ranged from 2.10% to 2.31%, NEAA (non-essential amino acids) content from 4.35% to 4.78%, and TAA (total amino acids) content from 6.45% to 7.09%. The order of EAA, NEAA, and TAA contents of foxtail millet under different fertilizer treatments was JN > JC > JCK > JP > JS. Compared with the control group, the EAA, NEAA, and TAA contents were significantly higher under the JN treatment, and the JC treatment was also favorable for the accumulation of EAA, NEAA, and TAA. On the contrary, the JS treatment significantly decreased the contents of each amino acid, and the JP treatment was also unfavorable for the accumulation of each amino acid. The trend of changes in the content of each amino acid component was the same as that of TAA; the EAA content of each fertilization group of LZGNo.2 ranged from 2.02% to 2.31%, the NEAA content from 4.16% to 4.81%, and the TAA content from 6.19% to 7.12%. Compared with the control group, the content of each amino acid increased to different degrees under ZC, ZN, and ZS treatments. The effect of nitrogen fertilizer on the content of each amino acid was better than other fertilization treatments, and there was a significant difference between ZN and other treatment groups. ZP treatment significantly reduced the content of each amino acid. Under different fertilization treatments, the content of phenylalanine in essential amino acids was the highest, and the content of tryptophan was the lowest.

### 2.4. Effects of Different Fertilizers on Starch Pasting Characteristics of Foxtail Millet

**Figure 2:**
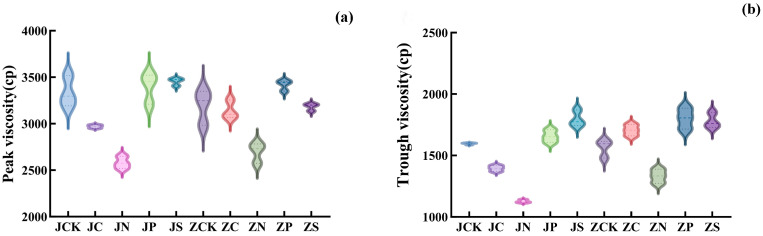

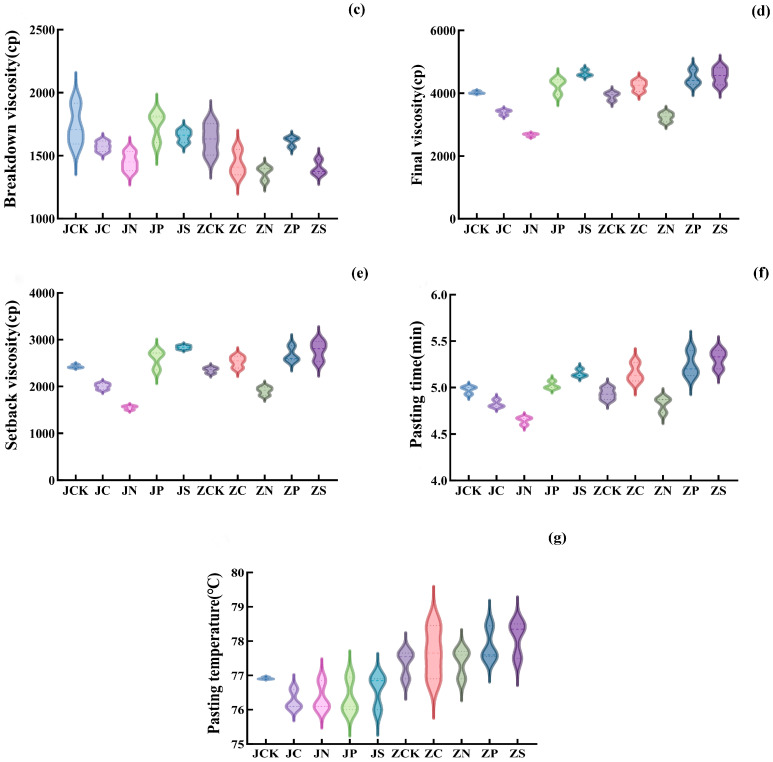
The effect of different fertilizer treatments on starch pasting characteristics: (**a**) Peak viscosity; (**b**) Trough viscosity; (**c**) Breakdown viscosity; (**d**) Final viscosity; (**e**) Setback viscosity; (**f**) Pasting time; (**g**) Pasting temperature. JN: JGNo.21 nitrogen fertilizer group; JP: JGNo.21 phosphorus fertilizer group; JC: JGNo.21 compound fertilizer group; JS: JGNo.21 organic fertilizer group; JCK: JGNo.21 control group, no fertilization; ZN: LZGNo.2 nitrogen fertilizer group; ZP: LZGNo.2 phosphorus fertilizer group; ZC: LZGNo.2 compound fertilizer group; ZS: LZGNo.2 organic fertilizer group; ZCK: LZGNo.2 control group, no fertilization.

In addition to the pasting temperature, other pasting parameters of JGNo.21 (Figure 2), such as peak viscosity, trough viscosity, breakdown viscosity, final viscosity, setback viscosity, and pasting time, had the same trend under different fertilizer treatments, and the JN and JC treatments significantly reduced the various pasting parameters, while the JP and JS increased the various pasting parameters compared with JCK. The variation in pasting temperature ranged from 76.27 °C to 76.92 °C. Different fertilization treatments reduced the pasting temperature, with the lowest pasting temperature under JC treatment. On the contrary, the pasting temperature of LZGNo.2 increased under each fertilizer treatment compared with the control, and the pasting temperature ranged from 77.37 °C to 78.12 °C, with the highest pasting temperature under the ZS treatment. The pasting parameters such as trough viscosity, final viscosity, setback viscosity, and pasting time showed the same trend under different fertilizer treatments, and compared with ZCK, ZC, ZP, and ZS treatments were able to improve the pasting parameters, and ZN reduced the pasting parameters. The peak viscosity was reduced under ZC, ZN, and ZS treatments, and ZP treatment significantly increased the peak viscosity. Breakdown viscosity was reduced under each fertilizer treatment.

### 2.5. Correlation Analysis

**Figure 3:**
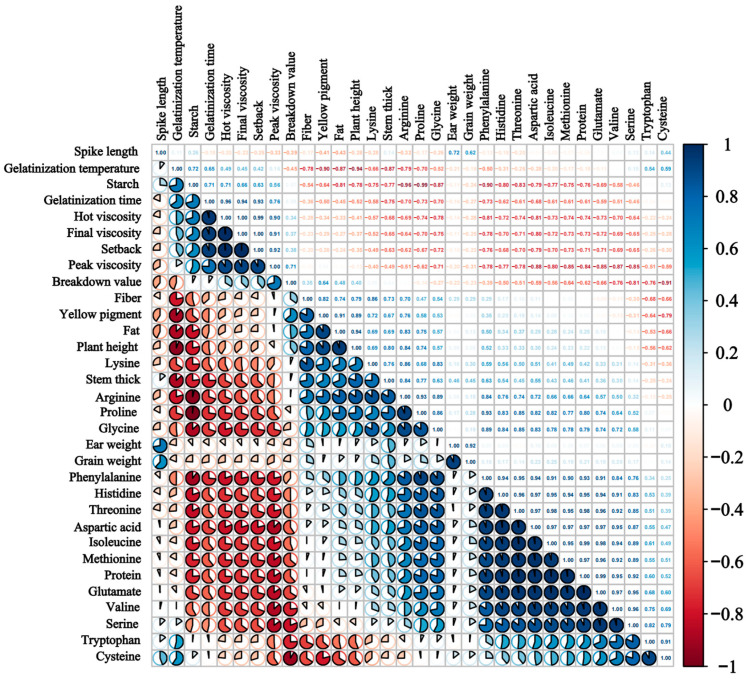
Correlation analysis of each index under different fertilizer treatments. The PC coefficient varies between −1 and +1; positive linear relationships between variables are highlighted in blue, and negative correlations are highlighted in red.

The correlation analysis of the measured agronomic traits, nutritional components, amino acid components, and starch pasting characteristics showed that there were various significant correlations between the indicators (Figure 3). Protein was significantly negatively correlated with starch, peak viscosity, trough viscosity, final viscosity, setback viscosity, breakdown viscosity, and pasting time, and significantly positively correlated with most amino acids. Among them, the positive correlation with isoleucine was the strongest (r = 0.99), and the negative correlation with peak viscosity was the strongest (r = −0.84). Fat was positively correlated with cellulose, yellow pigment, plant height, arginine, glycine, lysine, proline, phenylalanine, stem thickness, and breakdown viscosity, and negatively correlated with starch, pasting temperature, pasting time, tryptophan, and cysteine. Starch was significantly negatively correlated with fiber, yellow pigment, plant height, stem thickness, and most amino acid components, and significantly positively correlated with pasting parameters (except breakdown viscosity). Yellow pigment was positively correlated with plant height, stem thickness, breakdown viscosity, arginine, glycine, lysine, and proline, and negatively correlated with pasting temperature, pasting time, cysteine, and tryptophan. Plant height was significantly positively correlated with arginine, lysine, and proline. The breakdown viscosity was significantly negatively correlated with the pasting temperature. Other pasting parameters were significantly positively correlated with each other, and other pasting parameters were significantly negatively correlated with most amino acid components. Most of the amino acid fractions were strongly correlated with each other, with a highly significant positive correlation.

### 2.6. Principal Component Analysis

**Figure 4:**
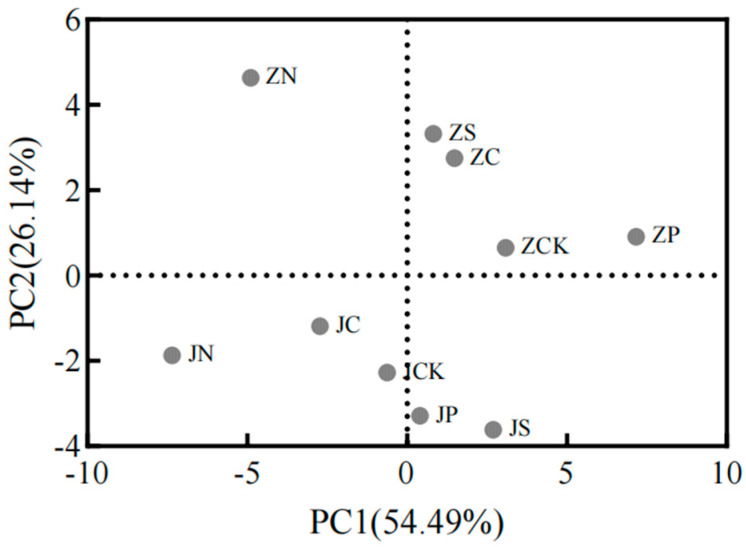
PCA scores of foxtail millet under different fertilizer treatments.

The principal component analysis (PCA) of the agronomic traits, nutrient components, amino acid components and starch pasting characteristics of foxtail millet under different fertilizer treatments showed that the eigenvalues of the first two principal components extracted by PCA were greater than 1, and the cumulative variance contribution rate was 80.63%, which was in line with the criterion of the variance contribution rate of 80% or more, and it could represent the majority of the information of the indexes measured, and could reflect the quality differences of foxtail millet under different fertilizer treatments accurately (Figure 4). The two PCs reflected the comprehensive grain quality under different fertilizer application modes in different aspects; the variance contribution rate of PC1 was 54.49%. Among them, starch contributed the most to PC1 (the load value was 0.892), followed by trough viscosity, final viscosity, peak viscosity, breakdown viscosity, and pasting time. PC1 mainly represents information on starch pasting characteristics. PC2, with a 26.14% variance contribution, mainly represents information on most of the variation in terms of amino acids. Separating the foxtail millet samples under different fertilizer treatments in the score plot, JP and JS were located in the positive direction of PC1 and the negative direction of PC2. JN, JC, and JCK were located in the negative direction of PC1 and the negative direction of PC2. ZN was located in the negative direction of PC1 and the positive direction of PC2. ZP, ZCK, ZC, and ZS were located in the positive direction of PC1 and the positive direction of PC2. According to the results of the principal component analysis, the quality of foxtail millet under JP, JCK, JC, and JS treatments was closer, and the quality of foxtail millet under ZP, ZCK, ZC, and ZS treatments was similar.

### 2.7. Systematic Clustering Analysis of Different Fertilizer Treatments

**Figure 5:**
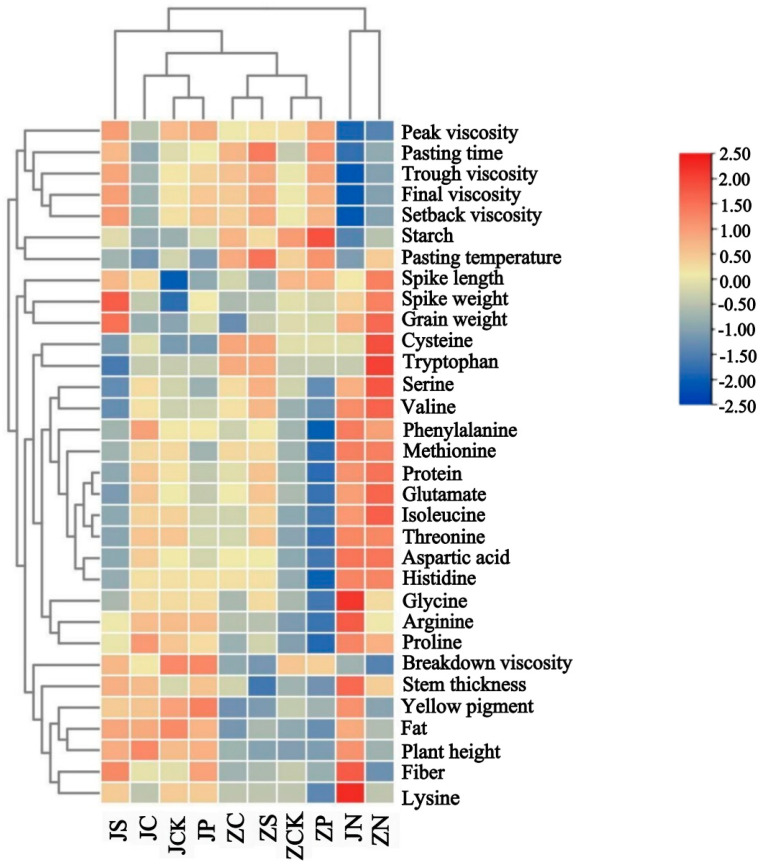
Cluster analysis heatmap of different fertilizer treatments and quality indicators.

Through the clustering analysis of different groups of fertilizer treatments and measured agronomic traits, nutritional components, amino acid components, and starch pasting characteristics indexes were found (Figure 5). The upper tree is the clustering of different groups of fertilizer treatments, and the different fertilizer treatments can be classified into four groups. Group I includes JS, JC, JCK, and JP, whose main characteristics are higher protein, fat, yellow pigment content, pasting parameter, and amino acid component. Group II consists of ZC, ZS, ZCK, and ZP, mainly characterized by lower protein, fat, fiber, yellow pigment, plant height, higher starch content, and lower pasting parameters and amino acid fractions. Group III is the ZN treatment, which is mainly characterized by high protein and amino acid fractions, better agronomic traits, low fat and fiber content, and low starch pasting parameters. Group IV is the JN treatment, which is mainly characterized by high protein, fat, and fiber content, higher plant height, low starch pasting parameters, and high amino acid fractions. The common characteristic of groups III and IV are low values of starch pasting parameters. The left tree shows the clustering of quality parameters, which are clustered into four groups. Group I includes pasting time, peak viscosity, trough viscosity, final viscosity, and setback viscosity. Group II includes starch and pasting temperature. Group III includes spike length, spike weight, and grain weight, and the rest of the quality indexes are all in Group IV, which includes breakdown viscosity, plant height, stem thickness, protein, yellow pigment, fat, cellulose, and amino acid fractions. The cluster analysis is similar to the PCA results, which can better reflect the differences in foxtail millet under different fertilizer treatments.

## 3. Discussion

### 3.1. Effect of Different Fertilizer Treatments on Agronomic Traits

Plant height determines whether grains can obtain more light energy in the population, and stem thickness is an indicator of grain lodging resistance. Both of them are closely related to the stem structure of grains. Lower plant height and thicker stem thickness can improve the overall lodging resistance of crops [28,29], while spike length, spike weight, and grain weight are closely related to yield [30]. Nitrogen application can significantly increase the photosynthetic pigment content of leaves, promote plant growth and development, increase leaf area, and delay leaf yellowing and senescence [31]. Zhang et al. [32] found that nitrogen application could prolong the grain filling period, thereby increasing grain weight. Nitrogen fertilization may be through modulation of photosynthesis, which in turn improves phenotypic traits in foxtail millet. In this study, nitrogen fertilizer can greatly improve plant height, spike length, stem thickness, spike weight, and grain weight traits compared to other fertilizers applied alone.

Phosphorus is an essential component for the formation of ATP, nucleic acids, proteins, and phospholipids, which are important components of photosynthesis; therefore, P deficiency affects plant photosynthesis [33]. P can accelerate photophosphorylation and enhance photosynthesis and the transfer of carbohydrates [34]. As the main source of soil phosphorus, phosphate fertilizer can increase the intensity of photosynthesis by accelerating photophosphorylation, enhancing the utilization of light energy, and promoting the accumulation of photosynthetic products. When nitrogen and phosphorus are applied at the same time, it can promote lateral root growth, and crop roots have stronger root signals, which is conducive to crop absorption of nutrients [35]. Under the treatment of phosphate fertilizer, plant height, spike length, stem thickness, spike weight and grain weight of foxtail millet increased.

Nadeem Shah et al. [17] showed that the application of organic manure favored increased leaf area. The larger the leaf area, the greater the photosynthesis and increased nutrient uptake through transpiration, which could improve panicle traits. The use of organic fertilizers increased soil porosity and significantly improved nutrient uptake by plants [17,36]. The mechanism of compound fertilizers on enhancing panicle traits is more inclined to be through improving nitrogen use efficiency and increasing leaf area index [19]. In the results of this study, compound and organic fertilizers had a positive regulatory effect on plant height, spike length, and stem thickness. Although different fertilizer treatments could improve all agronomic traits of JGNo.21, the response mechanism of LZGNo.2 to fertilizer was different from that of JGNo.21. Some agronomic traits were significantly different among different varieties, which may be due to genotypic differences or different sensitivities of different varieties to fertilizer.

### 3.2. Effect of Different Fertilizer Treatments on Nutritional Components and Amino Acid Composition

The quality of foxtail millet is not only dependent on genetic characteristics but also influenced by the ecological environment [37]. Therefore, fertilization can improve the environment of millet production, thereby improving the quality of millet. Whether the photosynthetic products of the grain-filling process are used for nitrogen or carbon metabolism depends on the ratio of sucrose phosphate synthase (SPS) to nitrate reductase (NR) activity [38]. Higher NR activity and glutamine synthase (GS) activity are conducive to nitrogen metabolism, that is, the synthesis of amino acids and proteins. During the early and middle stages of the irrigation process, the grain synthesizes protein by strong nitrogen transformation ability and by accumulating sufficient NH_4_^+^-N to supply amino acids, and higher GS activity allows NH_4_^+^ to be used for glutamine synthesis [39]. Glutamine translocation into the seed induces an increase in glutamate synthase activity, which leads to glutamate synthesis [40]. Wang et al. showed that increasing nitrogen application could increase the activity of NR and GS [41]. NR and GS promote nitrogen metabolism and produce glutamate and aspartic acid [42], which indicates that nitrogen fertilizer affects protein content by affecting nitrogen metabolism. In this study, nitrogen fertilizer can significantly increase protein content. The increase in protein content under organic and compound fertilizer treatments was not as good as that under nitrogen fertilizer treatment, which was the same as the results of Lou et al. [43]. When organic fertilizer completely replaced chemical fertilizer, the grain protein content decreased. The effect of different fertilizer treatments on the content of each amino acid was similar to that of protein, and the effect of nitrogen fertilizer on the content of each amino acid was also obvious.

Carbon metabolism includes the formation of sucrose, starch, and starch components, and the changes of enzyme activities related to starch synthesis [44]. Starch synthesis is regulated by a series of enzymes, among which ADP-glucan pyrophosphorylase (AGPase), granule-bound starch synthase (GBSS), starch branching enzyme (SBE), and soluble starch synthase (SSS) are essential enzymes in the synthesis process [45]. AGPase is the key and rate-limiting enzyme in starch synthesis. GBSS is mainly responsible for the synthesis of amylose, and the synthesis of amylopectin is synergistically driven by SSS, SBE, and debranching enzyme (DBE) [46]. The activities of SBE, AGPase, and GBSS were significantly or extremely significantly positively correlated with starch accumulation rate [39]. The study of Liu et al. [38] found that in wheat varieties with high starch accumulation, the kernel sucrose synthase (SS), phosphosucrose synthase (SPS), AGPase, SSS activities, and sucrose accumulation were higher than those of varieties with low starch accumulation, it is speculated that starch accumulation is affected by related enzyme activities and sucrose content. Nitrogen fertilizer affects the activity of key enzymes of starch synthesis, and thus, starch accumulation [47] and excessive nitrogen fertilizer has a negative effect on amylase activity [48]. Nitrogen fertilization significantly reduced starch content compared to the control. This indicates that nitrogen fertilizer may regulate starch content by affecting amylase activity. AGPase, the key enzyme in starch synthesis, is negatively inhibited by inorganic phosphate, so the activity and function of AGPase are affected by the content of P in plants [49]. Jiang et al. showed that the activities of GBSS, SSS, and SBE in wheat grains were the lowest at the grain formation stage and filling stage without phosphorus application [50]. In the present results, phosphorus fertilizer significantly increased the starch content, which corroborated the results of previous studies. In cereals, carbon and nitrogen metabolism utilize similar reducing power, ATP, and carbon skeleton [51]. Therefore, there is a competitive relationship between carbon and nitrogen metabolism, which explains the opposite effects of nitrogen and phosphorus fertilizers on protein and starch contents. Correlation analysis also showed that protein and starch contents were significantly negatively correlated.

The fat of foxtail millet consists of a variety of fatty acids and phytosterols, of which unsaturated fatty acids account for about 80% [52]. In the fatty acid metabolic pathway, acetyl-CoA carboxylase (ACCase) and 3-Ketoacy ACP reductase (FabG) are key enzymes in the synthesis process. Overexpression of ACCase and Fab G coding genes promotes the accumulation of fatty acids in foxtail millet [53]. In addition, ACCase activity is significantly affected by light energy utilization and chloroplast structure [54]. The application of different fertilizers can regulate photosynthesis [19,32,33,55]. Correlation analysis showed that fat content was significantly negatively correlated with starch content, which corresponded to the regulation of fertilizer on starch and fat content. The difference in the expression levels of ACCase and FabG genes is the reason for the difference in crude fat content between varieties [53], which explains the different response mechanisms of different varieties to fertilizer treatment from another perspective.

Sucrose is considered the initial substrate for cellulose biosynthesis; SS and SPS are also considered to be the key enzymes regulating cellulose biosynthesis [56]. The activities of SS and SPS are affected by light intensity; the weaker the light, the lower the enzyme activity, and the activity of SS and SPS is positively correlated with cellulose content [57]. Different fertilizer treatments can affect the light energy utilization efficiency of chloroplasts. Meanwhile, sucrose is the substrate of cellulose synthesis, but the sucrose content is correlated with the degradation of starch [46]. Therefore, the change in starch content can also be considered as a factor affecting cellulose content. Sitosterol in plant crude fat can be converted to sitosterol-β-glucoside, and sitosterol-β-glucoside can be used as a matrix for cellulose synthesis [58]. Therefore, we can speculate that cellulose content is regulated by many mechanisms, and further research is needed.

Yellow pigment is an important appearance quality of foxtail millet; the higher the content of yellow pigment, the yellower the color of foxtail millet. The main component of the yellow pigment is carotenoids [59]. The organelles that synthesize and store carotenoid metabolites are mainly located in chloroplasts and chromatin [60]. In chloroplasts, most carotenoids are mainly present in the form of pigment–protein complexes [61]. In addition, carotenoids belong to photosynthetic pigments [62], which indicates that the synthesis of carotenoids may be influenced by photosynthesis. Compared with the control, the yellow pigment content of JGNo.21 increased under nitrogen and phosphorus treatments, which may be due to the fact that nitrogen and phosphorus fertilizers can improve the photosynthetic capacity of crops [12,33], resulting in an increase in the pigment content that absorbs light energy.

### 3.3. Effects of Different Fertilizer Treatments on the Starch Pasting Characteristics

Starch pasting is the main change in the process of grain cooking. To clarify the process of starch pasting is helpful for us to understand the changes in food texture and structure [63]. The peak viscosity indicates the expansion degree of starch granules and the ability to bind water during gelatinization. When the peak viscosity is higher, the water-holding capacity of starch is better and easier to cook. The breakdown viscosity represents the degree of disintegration of starch during heating, reflecting the heat resistance and shear strength of starch. The higher the breakdown viscosity, the worse the stability; the setback viscosity indicates the retrograde characteristics of the starch paste after cooling. The higher the setback viscosity, the easier the retrogradation of starch paste after cooling. The pasting temperature and pasting time reflect the degree of difficulty of starch pasting; the higher the pasting temperature and the longer the pasting time, the more difficult it is to paste the starch [14,64].

Starch consists mainly of amylose and highly branched amylopectin [64]. Longer amylopectin chains can increase fluidity during retrogradation to form more intramolecular hydrogen bonds [65]. Hydrophilic functional groups enhance the water absorption capacity of starch, and these factors lead to higher pasting temperatures, peak viscosity, and breakdown viscosity. Changes in the length distribution of amylopectin are caused by changes in starch synthase activity, and starch branching enzymes play a key role in the elongation of long-chain amylopectin [66]. Wang and Singletary et al. have shown that nitrogen treatments can alter the activity of starch synthase [14,47]. Therefore, it is speculated that different fertilizer treatments regulate the distribution of short and long chains of amylopectin by changing the activity of starch synthase, ultimately affecting the pasting properties. In addition, the pasting characteristics are also affected by the protein content. From a microscopic point of view, it is found that with the increase in protein content, more proteins will adhere to the starch structure to form a honeycomb structure [67] and may bind to the surface of starch granules through spatial interactions, hindering starch water absorption and expansion, thus slowing down the process of starch pasting [68].

Compared with the control group, nitrogen fertilizer significantly affected the pasting properties of starch, as evidenced by the reduction in parameters such as peak viscosity, trough viscosity, final viscosity, breakdown viscosity, setback viscosity, pasting temperature, and pasting time. This is consistent with the results of Shi et al. [67], who found that nitrogen fertilizer could reduce the peak viscosity. Secondly, considering the significant increase in protein content under nitrogen fertilizer treatment and that protein–starch interaction hinders starch pasting [67,68], it is speculated that the reduction in viscosity parameters under nitrogen fertilizer treatment is also influenced by protein content. The peak viscosity, trough viscosity, final viscosity, breakdown viscosity, and setback viscosity of starch under phosphorus fertilizer treatment were higher than those of other treatment groups, which might be caused by the fact that phosphorus application could significantly increase the SBE activity [50], and the lower protein content under phosphorus fertilizer. The pasting characteristics of JGNo.21 responded differently to different fertilizer treatments. Nitrogen and compound fertilizers reduced the pasting viscosity, while phosphorus and organic fertilizers increased the pasting parameters. Phosphorus, compound, and organic fertilizers all increased the pasting viscosity of LZGNo. 2, and nitrogen fertilizer significantly reduced the pasting viscosity, which was opposite to the effect of fertilizer on protein content.

## 4. Materials and Methods

### 4.1. Experimental Design

The experiment was carried out in the Hequ Base of Agricultural Gene Resources Research Center of Shanxi Agricultural University in 2020. The experimental land was used for 4 consecutive years without nitrogen fertilizer. Phosphorus and potassium fertilizer were applied normally, and a foxtail millet/corn rotation was carried out. The soil nutrient conditions are as follows: pH value of 8.51, organic matter content of 8.16 g/kg, total nitrogen content of 0.61 g/kg, effective phosphorus content of 5.87 mg/kg, quick-acting potassium content of 97.07 mg/kg, and alkaline dissolved nitrogen content of 53.59 mg/kg. The meteorological data of the experimental site are shown in Table 3. The varieties of foxtail millet used for the experiment were JGNo.21 and LZGNo.2, which have a large planting area in the local production. The following five treatments were set up in the experiment: JN (urea, 10 kg/acres), JP (phosphate fertilizer, 8 kg/acres), JC (compound fertilizer, 25 kg/acres), JS (organic fertilizer, 2000 kg/acres), and JCK (control, no fertilizer) for JGNo.21; LZGNo.2:ZN (urea, 10 kg/acres), ZP (phosphate fertilizer, 8 kg/acres), ZC (compound fertilizer, 25 kg/acres), ZS (organic fertilizer, 2000 kg/acres), ZCK (control, no fertilizer). The N fertilizer used in the experiment was composed of urea (containing 46.4% pure N), the P fertilizer was composed of calcium superphosphate (containing 12.5% P_2_O_5_), the organic fertilizer was composed of sheep manure (containing 0.46% N, 0.29% P_2_O_5_, 0.24% K_2_O, 12.6% organic matter), and compound fertilizer was composed of NPK compound fertilizer (potassium phosphate nitrate ≥ 40%, N-P_2_O_5_-K_2_O: 17-20-4). The experiment was arranged in randomized blocks, with a plot area of 5 m × 6 m, six replications, and managed according to local crop cultivation practices.

### 4.2. Indicators and Methods of Determination

#### 4.2.1. Determination of Agronomic Traits

Fifteen uniform plants per plot were selected to measure the plant height, spike length, stem thickness, spike weight, and grain weight. Plant height was measured from the base of the stem to the longest tip of the tip in cm; spike length was measured from the node of the stem to the tip of the spike in cm; stem thickness was measured using vernier calipers to measure the diameter of the middle of the third internode above ground of the main stem of the grain in mm; spike weight was selected and weighed using an electronic balance in g; and grain weight was determined after threshing in g.

#### 4.2.2. Determination of Nutrient Fractions and Appearance Quality

The harvested grains were hulled with an experimental husker (BLH-3250, Taizhou, China). Hulled grains were ground into flour and passed through a 40-mesh sieve. Then, it was used to determine nutrient fractions and starch pasting characteristics. The protein content in foxtail millet was determined using the Kjeldahl nitrogen method [69]; some of the methods have been changed. Briefly, 0.5 g millet powder was weighed into the digestive tube, and then 0.5 g copper sulfate, 4.5 g potassium sulfate, and 10 mL concentrated sulfuric acid were added. The sample was digested at 420 °C for 1 h, and the completely digested liquid was green and transparent. The digested samples were automatically determined on an automatic Kjeldahl nitrogen analyzer (Kjeltec 8400, FOSS, Hillerød, Denmark). The total starch content was measured via anthrone colorimetry using a starch content kit (No. BC0700, Solarbio, Beijing, China). The fat content was determined using the Soxhlet extraction method [69]; some of the methods have been changed. A total of 2.0 g of millet flour was weighed and placed into a filter paper tube. Then, the filter paper tube was placed into the receiving bottle of the installed Soxhlet extractor (ST 255 Soxtec, FOSS, Hillerød, Denmark); the fat was extracted with petroleum ether, taken out of the receiving bottle, dried at 105 °C for 1 h, cooled to room temperature and weighed, and the fat content was calculated [69]. The crude fiber content was determined by the acid–base method using an automatic fiber work extraction (Fibertec 8000, FOSS, Hillerød, Denmark) [70]. Amino acid content was determined according to the method of Li et al. [71]. Briefly, HCl was added to the samples and hydrolyzed at 110 °C for 24 h. Two milliliters of the sample were filtered with a 0.2 μm needle filter (Minisart NY25, Sartorius, Göttingen, Germany), stored in vials, and loaded into an amino acid analyzer (S-433D, Sykam, Munich, Germany). Separation was performed using an ion exchange column. By comparing the area of amino acids in the sample and the standard, the amino acids in the sample were quantified. Yellow pigment content was determined by the method of Li et al. [71] with a slight modification: the absorbance of the extract was measured at 445 nm using a spectrophotometric method.

#### 4.2.3. Determination of Cooking Quality

The starch pasting curves of the samples were determined using a rapid viscosity analyzer with reference to the method of Wang et al. [14]. The suspension (3 g of sample, 14% water content, 25 mL of deionized water) was allowed to stand at 50 °C for 1 min, heated from 50 °C to 95 °C at 12 °C/min and held at 95 °C for 2 min. The suspension was then cooled to 50 °C at 12 °C/min and held for 1 min.

### 4.3. Statistical Analysis

Microsoft Excel 2021 software was used to statistically organize the data. One-way ANOVA, principal component analysis, correlation analysis, and other related statistical analyses were performed using OriginPro 2023 software, and the significance of differences between treatments was tested at the *p* < 0.05 level using the least significant difference (LSD) method, and the correlation was performed using Pearson correlation analysis, and graphing was performed using OriginPro 2023 software. All analyses had at least three replicates.

## 5. Conclusions

Different fertilization treatments affected agronomic traits, nutrient fractions, and pasting characteristics of foxtail millet to a certain extent. Nitrogen fertilizer had obvious improvement effect on various agronomic traits of foxtail millet. Phosphorus and organic fertilizer increased starch content, and nitrogen fertilizer had a greater effect on protein content. In addition, compound, nitrogen, and organic fertilizers also positively regulated essential, non-essential, and total amino acid content. On the other hand, the application of phosphorus and organic fertilizer could promote starch pasting. Under low nitrogen conditions, we systematically analyzed the effects of different fertilizer treatments on the agronomic traits, nutrient fractions, and pasting traits of foxtail millet by applying different fertilizer treatments to foxtail millet and clarified the regulation of different fertilizer types on the quality of foxtail millet under low nitrogen conditions. This provides a theoretical basis for rational fertilization and scientific fertilization and also provides a reference for improving the quality of foxtail millet.

## Figures and Tables

**Table 1:** Effects of different fertilizer treatments on agronomic traits.

Treatment	Plant Height/cm	Spike Length/cm	Stem Thickness/mm	Spike Weight/g	Grain Weight/g
JCK	146.11 ± 3.27 c	20.63 ± 0.94 a	7.11 ± 0.43 abc	14.75 ± 1.25 a	13.48 ± 1.82 ab
JN	158.78 ± 0.38 d	22.56 ± 0.25 ab	8.10 ± 0.34 c	19.31 ± 1.64 abc	15.75 ± 0.39 b
JS	150.78 ± 0.84 c	23.11 ± 1.26 ab	7.64 ± 0.65 bc	22.76 ± 2.80 c	16.79 ± 1.27 b
JC	161.89 ± 1.02 d	22.72 ± 0.54 ab	7.56 ± 1.05 bc	17.49 ± 0.39 ab	13.66 ± 1.10 ab
JP	149.70 ± 3.79 c	21.59 ± 0.80 ab	7.51 ± 0.90 abc	18.60 ± 2.02 abc	14.47 ± 1.58 ab
ZCK	108.78 ± 3.34 a	23.11 ± 2.01 ab	6.83 ± 0.33 ab	18.19 ± 2.73 abc	14.52 ± 2.02 ab
ZN	114.33 ± 2.96 b	23.78 ± 2.55 b	7.44 ± 0.53 abc	21.65 ± 4.33 bc	16.91 ± 3.39 ab
ZS	109.67 ± 3.93 ab	21.72 ± 2.86 ab	6.40 ± 0.84 a	17.32 ± 1.69 ab	14.27 ± 0.87 ab
ZC	114.67 ± 1.53 b	22.22 ± 1.17 ab	7.08 ± 0.34 abc	17.00 ± 4.06 ab	13.10 ± 2.27 a
ZP	108.33 ± 5.36 a	23.22 ± 0.69 ab	6.60 ± 0.24 ab	17.88 ± 1.05 ab	14.46 ± 1.26 ab
Variety	0.000 **	0.159	0.082 *	0.745	0.968
Treatment	0.488	0.601	0.971	0.333	0.838

Different letters in the same column indicate significant differences among treatments of different varieties (*p* < 0.05). * and ** respectively indicate significance at the 0.05 and 0.01 levels. JN: JGNo.21 nitrogen fertilizer group; JP: JGNo.21 phosphorus fertilizer group; JC: JGNo.21 compound fertilizer group; JS: JGNo.21 organic fertilizer group; JCK: JGNo.21 control group, no fertilization; ZN: LZGNo.2 nitrogen fertilizer group; ZP: LZGNo.2 phosphorus fertilizer group; ZC: LZGNo.2 compound fertilizer group; ZS: LZGNo.2 organic fertilizer group; ZCK: LZGNo.2 control group, no fertilization.

**Table 2:** Effects of different fertilization treatments on the content of amino acids.

Amino Acid		JCK	JC	JN	JP	JS	ZCK	ZC	ZN	ZP	ZS
EAA/(%) Essential amino acids	Thr	0.36 b	0.36 b	0.37 a	0.35 c	0.34 d	0.34 d	0.35 c	0.37 a	0.33 e	0.36 b
	Val	0.43 d	0.44 cd	0.46 b	0.43 e	0.41 f	0.42 e	0.44 d	0.47 a	0.41 f	0.45 c
	Trp	0.09 bc	0.09 bc	0.09 b	0.09 cd	0.08 d	0.09 b	0.10 a	0.11 a	0.09 bc	0.10 a
	Lys	0.19 b	0.18 bc	0.21 a	0.19 b	0.19 b	0.18 c	0.18 bc	0.18 bc	0.17 c	0.18 bc
	Phe	0.52 c	0.54 b	0.55 a	0.52 cd	0.50 ef	0.50 f	0.51 def	0.54 ab	0.47 g	0.52 cde
	Ile	0.34 cd	0.34 b	0.35 a	0.33 d	0.32 e	0.32 e	0.33 d	0.36 a	0.31 f	0.34 bc
	Met	0.26 b	0.26 b	0.27 a	0.25 cd	0.25 d	0.25 cd	0.26 c	0.27 a	0.24 e	0.26 b
NEAA/(%) Non-essential amino acids	Arg	0.36 b	0.36 b	0.38 a	0.36 bc	0.35 bc	0.33 ef	0.34 de	0.35 bc	0.32 f	0.34 cd
	His	0.20 bc	0.20 b	0.21 a	0.20 c	0.19 d	0.19 d	0.20 c	0.21 a	0.18 e	0.20 bc
	Asp	0.69 c	0.70 b	0.73 a	0.68 c	0.66 d	0.66 d	0.69 c	0.73 a	0.64 e	0.69 bc
	Cys	0.15 ef	0.16 cd	0.16 d	0.15 ef	0.15 f	0.16 bcd	0.17 bc	0.18 a	0.16 de	0.17 b
	Glu	1.81 c	1.85 bc	1.89 b	1.77 de	1.71 f	1.75 ef	1.81 cd	1.95 a	1.66 g	1.85 bc
	Gly	0.27 b	0.27 b	0.29 a	0.27 bc	0.26 cd	0.26 d	0.26 cd	0.27 b	0.25 e	0.27 bc
	Pro	0.66 bcd	0.68 ab	0.69 a	0.65 cde	0.64 de	0.60 g	0.61 fg	0.67 bc	0.57 h	0.63 ef
	Ser	0.41 d	0.42 bc	0.43 b	0.40 e	0.39 f	0.41 de	0.42 c	0.45 a	0.39 f	0.43 b
EAA		2.19 bc	2.21 b	2.30 a	2.16 d	2.09 e	2.10 e	2.17 cd	2.30 a	2.02 f	2.21 b
NEAA		4.55 bcd	4.64 b	4.78 a	4.48 d	4.35 e	4.36 e	4.50 cd	4.81 a	4.17 f	4.58 bc
TAA Total amino acids		6.74 bcd	6.85 b	7.08 a	6.64 d	6.44 e	6.46 e	6.67 cd	7.11 a	6.19 f	6.79 bc

JN: JGNo.21 nitrogen fertilizer group; JP: JGNo.21 phosphorus fertilizer group; JC: JGNo.21 compound fertilizer group; JS: JGNo.21 organic fertilizer group; JCK: JGNo.21 control group, no fertilization; ZN: LZGNo.2 nitrogen fertilizer group; ZP: LZGNo.2 phosphorus fertilizer group; ZC: LZGNo.2 compound fertilizer group; ZS: LZGNo.2 organic fertilizer group; ZCK: LZGNo.2 control group, no fertilization. Thonine: Thr; Valine: Val; Tryptophan: Trp; Lysine: Lys; Phenylalanine: Phe; Ieucine: Ile; Methionine: Met; Arginine: Arg; Histidine: His; Aspartate: Asp; Cysteine: Cys; Glutamic acid: Glu; Glycine: Gly; Proline: Pro; Serine: Ser; Different letters in the same row indicate significant differences between different treatments (*p* < 0.05).

**Table 3:** Meteorological data of foxtail millet growth period.

Month	Precipitation (mm)	Temperature (°C)			Mean Relative Humidity (%)	Average Wind Speed (m/s)	Duration of Daylight (h)
		Average	Highest	Lowest			
5	7.2	18.7	35.3	4.0	32	3.7	370.8
6	65.1	23.5	36.7	11.8	41	3.1	353.2
7	85.4	22.9	34.2	12.7	63	2.7	346.5
8	139.0	21.3	32.9	12.4	71	2.3	257.0
9	58.4	15.9	27.7	3.7	66	1.9	243.5
10	7.4	7.8	20.7	−5.2	47	2.2	259.2

## Data Availability

Data are contained within the article.
